# The impact of nurse’s sense of calling, organizational commitment, job stress, and nursing work environment on patient safety management activities in comprehensive nursing care service units during the covid-19 pandemic

**DOI:** 10.1186/s12912-024-01929-6

**Published:** 2024-05-07

**Authors:** YeJi Lee, Won Ju Hwang

**Affiliations:** 1 College of Nursing Science, Kyung Hee University, Seoul, South Korea; 2 College of Nursing Science, Kyung Hee University, East-west Nursing Research Institute, Seoul, South Korea

**Keywords:** Covid-19, Nurses, Patient safety

## Abstract

**Background:**

As the number of COVID-19 patients rises, there has been a notable increase in the workload for nurses. However, medium-sized hospitals lacked standardized protocols or consistent approaches to address the specific working conditions of nurses. Furthermore, concerns about patient care have heightened as the issue of nursing shortages coincides with the expansion of the comprehensive nursing care services project.

**Purpose:**

This study aimed to investigate the factors that influence patient safety management activities, such as calling, organizational commitment, job stress, and nursing work environment, among comprehensive nursing care service unit nurses during the COVID-19 pandemic.

**Methods:**

A conceptual framework based on the Job Demand-Resource model and literature review of patient safety management activities was used to develop structured questionnaires that were distributed to 206 participants working in 7 comprehensive nursing care service units of small and medium-sized hospitals with at least 300 beds in the S and K provinces. Data analysis was conducted using descriptive statistics, chi-squared tests, t-tests, ANOVA, and hierarchical regression with the SPSS/WIN 23.0 program.

**Results:**

The results showed that calling (β =.383, p<.001) and job stress (β= -.187, *p*=.029) significantly influenced patient safety nursing activities in comprehensive care service ward nurses. The explanatory power of the model was 26.0% (F= 6.098, *p*<.001).

**Conclusions:**

Our findings suggest that comprehensive care service ward nurses' career, income, COVID-19 patient nursing anxiety, calling, and job stress were important factors that influence patient safety nursing activities. Therefore, it was essential to develop calling education programs and improve the nursing work system and establish a fair compensation system during the pandemic situation.

## Introduction

### Background

In February 2021, the COVID-19 vaccine was introduced in South Korea, and vaccination was carried out starting with healthcare professionals and then vulnerable populations. Despite the increasing vaccination rates, the number of new COVID-19 cases continued to rise, with over 1,000 new cases per day since July 2021, leading the government to declare a "fourth wave" of the pandemic [1]. During the early stages of the COVID-19 outbreak, there was confusion in hospitals regarding the screening and admission process for COVID-19 patients, which led to the revision of the Infection Management Medical Law, resulting in nurses strictly adhering to respiratory infection control, standard precautions, and the use of personal protective equipment to focus on patient safety [2]. However, as the COVID-19 situation has continued long-term, studies have suggested a reevaluation of the "heroic" image of nurses under social pressure.

Comprehensive nursing care services aim to provide high-quality nursing care, with improvements in patient safety such as prevention of falls, pressure ulcers, medication administration, and infection control emphasized in medical institution evaluations. However, the expansion of comprehensive nursing care service units coincided with the COVID-19 situation, resulting in an increased workload for nurses in these wards. Therefore, due to the close relationship between the increased direct nursing activities and patient safety nursing activities in existing comprehensive nursing care service units, and the increased sense of responsibility for patient safety nursing activities due to the COVID-19 situation, it is necessary to explore methods to improve the quality of patient safety nursing activities by nurses in these wards.

Sense of calling is a mindset that goes beyond religious meaning and involves finding fulfillment in one's work and making socially significant contributions, recognizing the value of giving meaning to one's work and contributing to society [3]. Those who possess a sense of calling not only find satisfaction in their work, but also gain the strength to persevere through various obstacles [4]. Following the COVID-19 situation, the media has shown interest in nurses who care for patients with a sense of calling, but there has been a lack of diverse activities and accurate behavior descriptions of nurses [5]. However, the professional ethics of nurses who worked at COVID-19 screening clinics to prevent the spread of infectious diseases during crisis situations was a sense of calling [6], which can be seen as the driving force behind patient safety nursing activities.

Organizational commitment refers to an individual's willingness to work for and remain a member of the organization to which they belong and is an important variable that promotes efficient task performance among members, ultimately enhancing the organization's performance and quality of nursing services [7]. Particularly during outbreaks of novel infectious diseases, securing personnel and fostering close cooperation among medical personnel are essential to respond effectively [8]. Therefore, identifying factors that contribute to patient safety nursing activities, such as organizational commitment, is not only important for clinical nurses, but also necessary for the expansion and development of comprehensive nursing care services in small and medium-sized hospitals.

Implementation of comprehensive nursing care services has been reported to increase patient satisfaction and hospital reutilization rates compared to general hospital wards [9]. However, it also leads to an increase in job stress for nurses due to demands from patients who expect nursing services beyond their scope of work [10]. Additionally, it results in increased documentation for tasks like skin care, medication management, and more, adding to job stress [11] comprehensive nursing care services wards have nurses who constantly enter and leave patient rooms depending on their demands, as there is no primary caregiver staying with the patients. Given the easy transmission of COVID-19 through close contact, comprehensive nursing care service unit nurses are stressed by the risk of exposure to infection sources [12]. Therefore, nurses in comprehensive nursing care services wards are facing a double burden of existing workload and new expectations and responsibilities related to COVID-19 prevention measures.

The nursing work environment encompasses various aspects, including physical, relational, and managerial characteristics that enable nurses to provide professional care [13]. When comparing small and large hospitals operating comprehensive nursing care service units, small hospitals face difficulties in securing nursing staff due to lower salaries and poor working conditions, leading to a concentration of nursing personnel in large hospitals [14]. Thus, there is an increasing demand for safe and secure nursing services from hospital customers [15], while small hospitals' nursing staff shortages can lead to difficulties in providing quality nursing services.

**Fig. 1:**
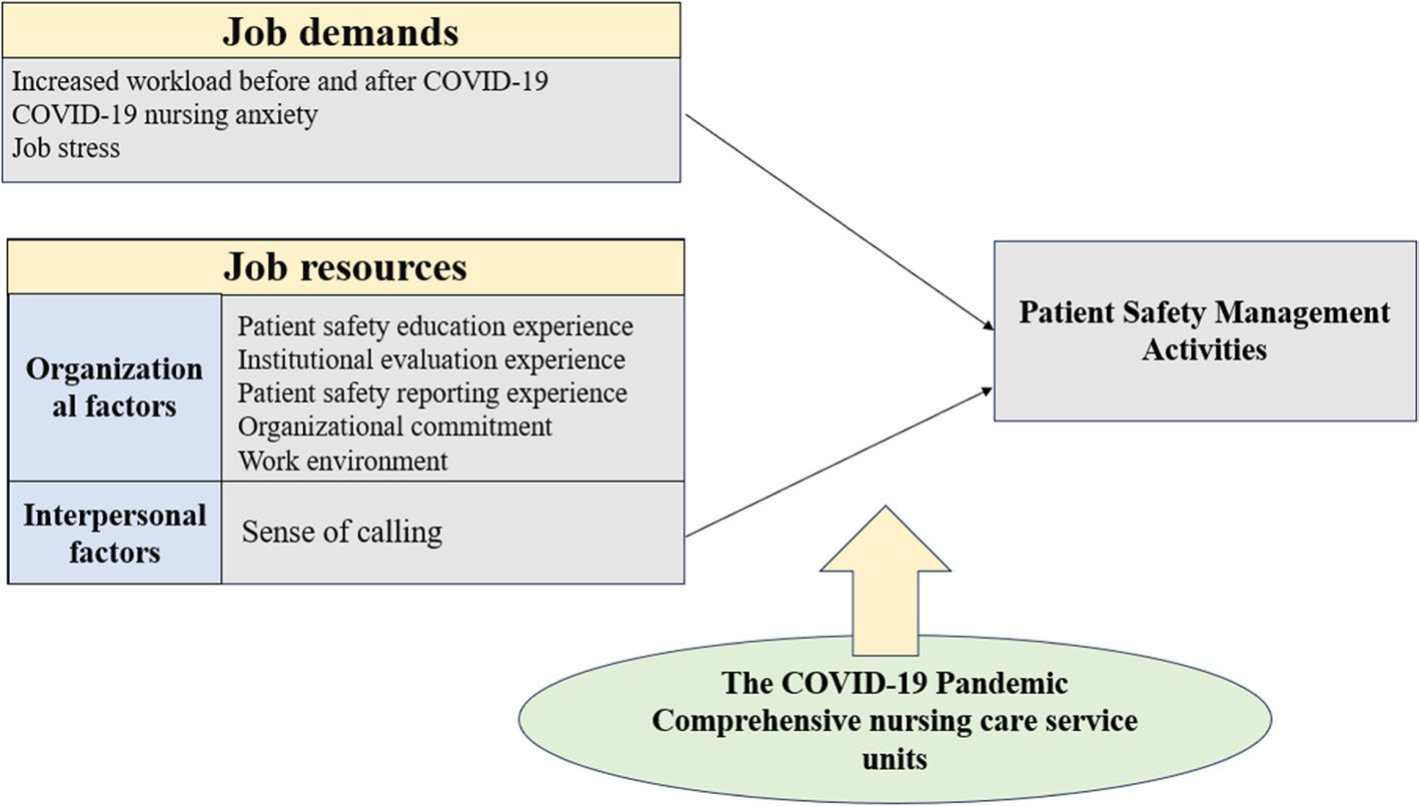
Research model applying the job demands-resources model

Likewise, the COVID-19 pandemic broke out during the expansion of the comprehensive nursing care service project in small and medium-sized hospitals and nurses were working with sense of calling in this chaotic nursing environment. However, because there were no approved work guidelines and protocol for nurses, nursers had to bear work stress, so they left the field and threatened patient safety. Therefore, this study attempted to identify the relationships between nurses' sense of calling, organizational commitment, job stress, and nursing work environment, and the factors influencing patient safety nursing activities among nurses in small hospitals' comprehensive nursing care service units. The conceptual framework of this study was grounded in the Job Demand Resource (JD-R) Model proposed by Bakker and Demerouti [16], upon which the following research model was constructed (Fig.  1).

### Purpose

The purpose of this study was to identify the factors affecting patient safety nursing activities in the context of COVID-19 by investigating the relationship between the sense of calling, organizational commitment, job stress, and nursing work environment of nurses in small and medium-sized hospitals' comprehensive nursing care service units. To achieve this, the study aimed to examine the general characteristics, level of sense of calling, organizational commitment, job stress, nursing work environment, and patient safety nursing activities of nurses in comprehensive nursing care service units. Based on these findings, the study aimed to provide information on the relationship between sense of calling, organizational commitment, job stress, nursing work environment, and patient safety nursing activities, as well as the factors influencing this relationship.

## Methods

### Study design

The present study was a descriptive survey research designed to investigate the level of general characteristics, sense of calling, organizational commitment, job stress, and nursing work environment of nurses working in small and medium-sized hospital nursing care service units, and to identify their impact on patient safety nursing activities. Prior to the start of the study, approval was obtained from the Institutional Review Board (IRB) of K University under the reference number (KHSIRB-21-583).

### Participants

The participants of this study were from seven small to medium-sized hospitals in the S and G metropolitan areas, each with a comprehensive nursing care service unit consisting of more than 100 beds and less than 300 beds. One of the hospitals was a specialized spinal joint hospital, while the other six were general hospitals. Since the COVID-19 outbreak, three hospitals have been operating COVID-19 residential treatment centers and dispatching nurses, two hospitals have been operating COVID-19 inpatient wards, and two hospitals have been monitoring COVID-19 patients who are under home quarantine. Newly employed nurses with less than one year of clinical experience were excluded as they were in a period of adjusting to the hospital organization, which could lead to decreased organizational commitment, based on previous studies [17]. The sample size of the participants was calculated using G*power 3.1, and the study targeted a total of 206 participants, considering a dropout rate, with 183 participants required for a regression analysis to explain the study's power with an effect size of 0.15, 18 independent variables, a significance level of 0.05, and a test power of 0.9. Sixteen questionnaires with unreliable responses were excluded, resulting in 190 questionnaires being used for analysis.

### Sampling method

This study gathered data from February 24, 2022, to March 3, 2022, at seven small and medium-sized hospitals with nursing and nursing integrated service wards, each having 100 to 300beds. These hospitals have situated in S City and the Gyeongin region. The target demographic for the data collection was nurses, and an online survey was employed for this purpose. Owing to COVID-19 quarantine protocols, external visitors had not allowed at the hospitals. Therefore, the study's objectives and details were communicated to the nursing department over the phone, and explicit permission to collect data was obtained. The researcher provided the online link to the nursing department, and through the unit manager, the department distributed an online link to the structured mobile questionnaire to those who willingly agreed to participate in the study. The research subject's consent ensures that personal information was kept confidential during their participation in the research, granting them the right to refuse involvement even after the research has started. The research results were kept anonymous and will be exclusively utilized for research purposes. These findings were intended for both statistical purposes and potential publication. The process begins with a research consent form explaining that the survey can be conducted, and only those who have confirmed their consent online before the survey were directed to the survey participation. To deter fictitious participation in the web-based survey process, participants were required to respond to all items during the survey, and each question was configured to allow only one response to prevent multiple submissions. In cases where additional clarification was necessary, participants had the option to receive explanations over the phone. As compensation for the time taken due to participation in this study, a gift (online beverage voucher) was provided. Prior to delivering the gift, phone numbers collected before sending it were checked for duplicate participation, and phone numbers collected after the confirmation of sending the gift were discarded.

### Demographic and work characteristics

Nurse’s Demographic and work characteristics were obtained by survey. Demographic and work variables included in the study were participants’ age, education level, work experience, income, increased workload after COVID-19, COVID-19 patient nursing anxiety, patient safety education experience, and institutional evaluation.

### Measures

1. Patient Safety Nursing Activities

Patient safety nursing activities were measured using the tool developed by Park [18]. The tool consists of 24 items categorized into six domains: patient identification (4 items), communication among healthcare providers (5 items), high-risk medication management (2 items), accurate surgery and procedure verification (3 items), infection prevention (7 items), and fall prevention (3 items). Each item was rated on a 5-point Likert scale ranging from "never" (1) to "always" (5), with higher scores indicating higher levels of patient safety management activities. The tool demonstrated good reliability with a Cronbach's α of .87.

2) Sense of Calling

The Multidimensional Measure of Calling (MCM) developed by Hagmaier and Abele [19] was used to measure calling, which was adapted to Korean by Ha et al.[20]. The Korean version of the tool consists of 9 items, categorized into three dimensions: job-person fit and environmental fit (3 items), meaning and value pursuit (3 items), and transcendent summons (3 items). Each item was rated on a 6-point Likert scale ranging from "not at all" (1) to "very much" (6). The tool demonstrated good reliability with a Cronbach's α of .88.

3) Organizational Commitment

The measurement tool for organizational commitment used in this study was a three-factor organizational commitment scale that was validated for Koreans by Lee et al. [21], based on the organizational commitment tool developed by Meyer and Allen [22]. The scale consists of three sub-factors: affective, continuance, and normative commitment, and each item was measured on a 7-point Likert scale ranging from "strongly disagree" (1) to "strongly agree" (7). The reliability of the scale was confirmed with a Cronbach's α of .93.

4) Job Stress

The measurement tool for job stress used in this study was the Effort-Reward Imbalance (ERI) tool developed by Siegrist et al. [23]. The translation was confirmed by two bilingual experts who evaluated its linguistic and cultural suitability. Job stress was assessed by the validated Korean version of the original ERI questionnaire containing 23 items [23, 24]. The reported internal consistency for the Korean version was satisfactory with 0.71 for effort and 0.86 for reward [24]. The effort and reward domains each have five items measured on a 5-point Likert scale, with the minimum score for effort being 6 and the maximum score being 30, while the minimum score for reward was 11 and the maximum score was 55. To analyze job stress, the ratio of the sum of effort items to the sum of reward items was calculated. As the number of items for effort and reward domains was different (6 and 11, respectively), the total score for the reward domain was multiplied by 6/11 = 0.545 to make the maximum score 1. A ratio of 1 indicates a balance between effort and reward, while a ratio less than 1 suggests that rewards were greater than the subjective effort, indicating no job stress, and a ratio greater than 1 indicates that rewards were lower than the subjective effort, indicating job stress.

Over commitment refers to excessive dedication to excessive work demands, with higher scores indicating more commitment due to excessive control demands. The scale for over commitment was measured on a 4-point Likert scale ranging from 6 to 24, with higher scores indicating greater commitment due to excessive control demands. The reliability of the scale was Cronbach's α of .71.

5) Nursing Work Environment

The nursing work environment was measured using the Korean version of the nursing work environment scale developed by Lake[25] and validated by Cho et al.[26] This scale consists of 29 items, organized into five sub-domains: patient surveillance (4 items), interprofessional communication (5 items), high-risk medication management (2 items), accurate procedure verification (3 items), infection prevention (7 items), and fall prevention (3 items). Each item was rated on a 4-point Likert scale ranging from "strongly disagree" (1) to "strongly agree" (4), with higher scores indicating more positive perceptions of the nursing work environment by the nurses. The reliability coefficient was Cronbach's α of .93.

### Data collection

From February to March 2022, an online survey was conducted targeting nurses working in seven small to medium-sized hospitals in S city and the Gyeongin area that operate comprehensive nursing care service units with 100 to 300 beds. One of the seven institutions was a specialized hospital for spinal joint care, and the others were general hospitals with more than seven essential treatment departments. All medical institutions were private hospitals, and the operation of comprehensive nursing care service units began in 2017. After the COVID-19 outbreak, three of the institutions operated hospital beds for COVID-19 infected patients, and the rest were engaged in running care centers, dispatching personnel to COVID-19 dedicated hospitals, and monitoring COVID-19 high-risk individuals in self-quarantine. As per the COVID-19 prevention guidelines, external visitors were prohibited from entering the hospital, so the researchers explained the purpose and content of the study to the nursing staff over the phone and received permission for data collection. The researchers provided an online link to the nursing staff, who then provided a structured mobile survey link (Google) to the voluntary participants via the nursing staff. Participants received a compensation of a reasonable reward (online beverage voucher) for their participation in the study.

### Statistical analysis

This study used SPSS Statistics 23.0 as the analytical tool for research problem and hypothesis testing. General characteristics of subjects were analyzed using descriptive statistics such as frequency, percentage, and mean. The level of significance of nursing job stress was analyzed by chi-squared test, and the means and standard deviations of sense of calling, organizational commitment, nursing job stress, nursing work environment, and patient safety nursing activities were calculated using descriptive statistics. Differences in patient safety nursing activities according to general characteristics were analyzed using t-tests and ANOVA, and post hoc tests were conducted using Scheffé test. Pearson's correlation coefficient was used to analyze the relationships between sense of calling, organizational commitment, nursing job stress, nursing work environment, and patient safety nursing activities. Finally, the hierarchical regression analysis was used to examine the effects of sense of calling, organizational commitment, nursing job stress, and nursing work environment on patient safety nursing activities.

## Results

### Differences in patient safety nursing activities according to the general characteristics of study participants

The demographic characteristics of the participants are presented in Table 1. Regarding the differences in patient safety nursing activities according to general characteristics, significant differences were found in the comprehensive nursing care service unit work experience (F=5.426, *p*<.001), income (F=3.805, *p*=.011), anxiety related to caring COVID-19 patients (F=2.795, *p*=.028), and institution evaluation experience (t=2.325, *p*=.021). In terms of the comprehensive nursing care service unit work experience (F=5.426, *p*<.001), those with less than 2 years of experience had higher patient safety nursing activity scores than those with 3-4 years of experience. Regarding income (F=3.805, *p*=.011), those with a monthly income of 351 million won or more had a higher patient safety nursing activity score, and a post-hoc test showed a significant difference between the income groups of 250 million won, 251-300 million won, 301-350 million won, and 351 million won or more. Anxiety related to nursing COVID-19 patients (F=2.795, *p*=.028) showed higher scores for those who reported "yes" (110.18±9.98 points), but there was no significant difference in the post-hoc test. Institution evaluation experience (t=2.325, *p*=.021) was associated with higher patient safety nursing activity among those who reported having evaluation experience. Educational level, work experience, position, increased workload after COVID-19, patient safety incident experience, and patient safety education experience within the past year were not significant (Table 1).

**Table 1:** Comprehensive Nursing Care Service Ward Nurse's Characteristics**.** (*N*=190)

Characteristics	Categories	n(%)	Patient Safety-management activities		
			M±SD	t or F (p)	Scheffe
Age	20s	84(44.2)	107.95±8.25	0.493(.612)	
	30s	45(23.7)	107.38±9.87		
	$\ge$40s	61(32.1)	109.10±10.12		
Average Age	34.9				
Education level	3-year diploma	57(30.0)	108.09±10.65	0.005(.995)	
	Bachelor	126(66.3)	108.23±8.61		
	$\ge$master	7(3.7)	108.14±9.51		
Work experience(yr)	1~3	25(13.2)	111.40±8.31	2.055(.108)	
	3~5	54(28.4)	106.63±8.27		
	5~10	41(21.6)	106.80±8.29		
	$\ge$10	70(36.8)	109.04±10.51		
Work experience in current job(yr)	$<$1^a^	26(13.7)	110.54±8.85	5.426(<.001^***^)	d<b
	1~2^b^	28(14.7)	112.68±7.55		
	2~3^c^	41(21.6)	109.17±8.98		
	3~4^d^	60(31.6)	104.22±8.88		
	4~5^e^	35(18.4)	108.49±9.55		
Job position	Staff nurse	132(69.5)	107.47±9.54		
	$\ge$Charge nurse	58(30.5)	107.84±7.75	2.859(.06)	
Income(10,000KW/M)	$\le$250^a^	12(6.3)	100.33±15.02	3.805(.011^*^)	a<b,c,d
	251~300^b^	88(46.3)	108.17±8.72		
	301~350^c^	69(36.3)	108.68±8.07		
	$\ge$351^d^	21(11.1)	111.10±9.22		
Increased workload after COVID-19	NO	27(14.2)	108.93±9.76	-0.449(.654)	
	YES	163(85.8)	108.06±9.19		
COVID-19 patient nursing anxiety	SD	59(31.1)	109.25±7.64	2.795(.028^*^)	-
	D	62(32.6)	110.18±9.98		
	N	42(22.1)	105.81±9.97		
	A	25(13.2)	105.48±7.99		
	SA	2(1.1)	98.50±13.44		
Patient safety accident report experience	NO	47(24.7)	109.38±11.23	-0.892(.376)	
	YES	143(75.3)	107.79±8.51		
Patient safety Education experience (within an yr)	NO	13(6.8)	104.23±10.35	1.604(.110)	
	YES	177(93.2)	108.47±9.13		
Institutional evaluation experience	NO	95(50.)	106.64±10.01	2.325(.021^*^)	
	YES	95(50.0)	109.73±8.18		

^*^p<.05

^**^p<.01

^***^p<.001

Scheffé test (a < b). *M* Mean, *SD* Standard deviation

### Degrees of sense of calling, organizational commitment, job stress, work environment, patient safety management activities

The overall average score of the participants' sense of calling, based on the nine questions, was 4.35 ± 0.85, with the highest average score in the area of the meaning and pursuit of work at 4.53 ± 0.91. The overall average score of the participants' organizational commitment, based on 18 questions, was 3.57 ± 1.17, with the highest average score in the normative sub-factor area at 3.63 ± 1.13. The participants' job stress was measured by 17 questions, with sub-factor areas of effort (3.58 ± 0.68), reward (1.74 ± 0.31), and the ratio of effort to reward ranging from a minimum of 0.51 to a maximum of 2.67, with an overall average score of 1.17 ± 0.35, which was higher than 1. Over commitment was measured by six questions, with an average score of 2.57 ± 0.60. The average score for the nursing work environment was 2.27 ± 0.46, with the highest average score in the area of collaboration between nurses and doctors at 2.44 ± 0.63, and the lowest average score in the areas of nursing manager's ability, leadership, and support for nurses at 2.14 ± 0.62. The average score for the participants' patient safety nursing activities was 4.77 ± 0.42, with the highest score in the area of fall prevention at 4.66 ± 0.46, and the lowest score in the area of high-risk medication management at 4.01 ± 0.76 (Table 2).

**Table 2:** Degrees of Calling, Organizational commitment, Job stress, Work environment, Patient safety management activities (*N*=190)

Variables	Categories	Number of items	Range	
				M±SD
Calling		9	14~36	4.35±0.85
organizational commitment	Affective	18 6	21~126 7~42	3.57±1.17 3.50±1.52
	Continuance	6	6~42	3.56±1.28
	Normative	6	6~42	3.63±1.13
Job stress	Effort Reward Overcommitment	23 6 11 6	0.51~2.67 10~30 6~30 7~25	1.17±0.35 3.58±0.68 1.74±0.31 2.57±0.60
Work environment		29 9	35~115 9~36	2.27±0.46 2.19±0.54
Patient safety management activities	Identification of patient Communication of staff High-risk medication nursing Accurate surgery and procedure check	24 4 5 2 3	84~120 12~20 9~25 6~10 9~15	4.77±0.42 4.51±0.39 4.61±0.45 4.01±0.76 4.55±0.61
	Infection	7	21~35	4.55±0.51
	Falls	4	9~15	4.66±0.46

*IP* Identification and P-E Fit, *SMVB* Sense of Meaning and Value-driven Behavior, *TGF* Transcendent Guiding Force

### Association with job stress among study participants

In addition, a cross-analysis was conducted to identify variables associated with job stress among study participants. The analysis results showed that there was an association between age group and increased workload after the COVID-19 outbreak, with χ2 =9.089, *p*=.011. This suggests that there was a correlation between job stress and age group. The highest frequency of job stress was reported among individuals in their 20s, with 38.3% (49 individuals) reporting job stress. There was also an association between job stress and increased workload after the COVID-19 outbreak, with χ2 =7.523, *p*=.006. Job stress was reported as "yes" by 90.6% (116 individuals) of participants who experienced increased workload after the COVID-19 outbreak, which was the highest frequency reported (Table 3).

**Table 3:** Job stress among study participants (*N*=190)

Variables		n(%)		x^2^(p)
		1 < ERI	1 > ERI	
Age				9.09(.011)
	20s	35(56.5)	49(38.3)	
	30s	7(11.3)	38(29.7)	
	$\ge$ 40s	20(32.3)	41(32.0)	
Increased workload after Covid-19				7.52(.006)
	NO	15(24.2)	12(9.4)	
	YES	47(75.8)	116(90.6)	

### The correlation between nurses’ sense of calling, organizational commitment, job stress, nursing work environment, and patient safety management activities

The correlation between nurses' general characteristics, sense of calling, organizational commitment, job stress, working environment, and patient safety management activities were examined. Patient safety management activities showed a static correlation with sense of calling (r=.04), organizational commitment (r=.16), and working environment (r=.20). This means that higher sense of calling, organizational commitment, and better working environment led to higher levels of patient safety management activities. There was a significant negative correlation between job stress and patient safety nursing activities (r=-.19), indicating that higher job stress leads to lower patient safety nursing activities (Table 4)

**Table 4:** Correlation among Research Variables (*N*=190)

Variables	Patient safety management activities:	Identification of patient	Communi-cation of staff	High-risk medication nursing	Accurate surgery and procedure check	Infection	Falls
	r (*p)*	r (*p)*	r (*p)*	r (*p)*	r (*p)*	r (*p)*	r (*p)*
Calling	.40 (<.001)	.32 (<.001)	.29 (<.001)	.31 (<.001)	.38 (<.001)	.30 (<.001)	.09
Organizational commitment	.16(.029)	.08	.18(.011)	.08	.20(.007)	.11	-.10
Job stress	-.19(.011)	-.06	-.23(.001)	-.02	-.12	-.12	-.11
Nursing work environment	20(.007)	.05	.29(,.001)	-.01	.18(.012)	12	-.03

### Factors in patient safety management activities

Factors affecting patient safety nursing activities of the participants were found in Table 5. To identify the factors influencing patient safety nursing activities of the study participants, with patient safety nursing activities as the dependent variable, independent variables such as comprehensive nursing care service unit experience, income, COVID-19 nursing anxiety, institutional evaluation experience, sense of calling, organizational commitment, job stress, and nursing work environment were selected from the general characteristics with significant differences in patient safety nursing activities. Before conducting the regression analysis, multicollinearity diagnosis was performed, and the VIF (Variance Inflation Factor) ranged from 1.064 to 2.579, all of which were less than 10, and the tolerance limits ranged from .39 to .94, all of which were between 0.1 and 1.0, indicating that there was no problem of multicollinearity in the data. The Durbin-Watson test result was 1.961, indicating that there was no multicollinearity and that the residual distribution satisfied the normality assumption. In the results of residual analysis, all assumptions of linearity, normality of errors, and homoscedasticity were satisfied. There were no outliers.

**Table 5:** Influencing Factors in Patient Safety Management Activities (*N*=190)

Variables	Model1				Model2					Model3			
	B	SE	*β*	t(p)	B	SE	*β*	t(p)		B	SE	*β*	t(p)
Age	-.08	.66	-.01	-.12(.902)	-.11	.64	-.01	-.17(.865)		-.84	.65	-.09	-1.30(.197)
Education level	-1.17	1.29	-.07	-.91 (.364)	-1.84	1.28	-.10	-1.44(.153)		-2.17	1.22	-.12	-1.78(.076)
Work experience in current job	-1.92	.512	-.27	-3.69(<.001)	-1.68	.53	-.24	-3.14 (.002)		-1.48	.52	-.21	-2.87(.005)
Income	3.05	.90	.26	3.38(.001)	2.67	.90	.22	2.99 (.003)		2.62	.86	.22	3.06(.003)
Covid-19 patient nursing anxiety					-1.59	.61	-.18	-2.61 (.010)		-1.34	.59	-.15	-2.27(.024)
Patient safety report experience					-2.70	1.64	-.13	-1.65(.100)		-1.08	1.59	-.05	-.68(.496)
Patient safety education experience					4.01	2.56	.11	1.57(.118)		2.83	2.46	.08	1.15(.251)
Institutional evaluation experience					3.44	1.28	.19	2.68 (.008)		1.93	1.30	.10	1.48(.140)
Calling										.58	.15	.32	3.94(<.001)
Organizational commitment										.03	.10	.03	.31(.761)
Job stress										-4.54	2.29	-.17	-1.98(.049)
Nursing work environment										-.03	.06	-.05	-.48(.632)
*F(p)*=5.24(.001)					*F(p)*=4.87(<.001)				*F(p)*=5.78(<.001)				
R^2^=.10					R^2^=.18				R^2^=.30				
Adj.R^2^=.08					Adj.R^2^=.14				Adj.R^2^=.26				

In Model 1, since most demographic variables among the general characteristics have a relationship with social phenomena, age and education were used as control variables to minimize bias in the regression coefficient estimates. When comprehensive nursing care service unit experience and income, which showed statistically significant differences in the difference analysis, were entered, the regression model was statistically significant (F=5.243, *p*<.001). In Model 2, when COVID-19 nursing anxiety and patient safety-related characteristics were additionally included, the regression model was statistically significant (F=4.874, *p*<.001), and comprehensive nursing care service unit experience (β=-.235, *p*=.002), income (β=.224, *p*=.003), COVID-19 nursing anxiety (β=-.182, *p*=.01), and institutional evaluation experience (β=.182, *p*=.008) were significant influencing factors, and the explanatory power of the model increased by 17.7%.

In Model 3, when sense of calling, organizational commitment, job stress, and nursing work environment were additionally included, the regression model was statistically significant (F=6.098, *p*<.001), and the comprehensive nursing care service unit experience (β=.209, *p*=.004), income (β=.241, *p*=.001), COVID-19 nursing anxiety (β=-.134, *p*=.048), sense of calling (β=.383, *p*<.001), and job stress (β=-.187, *p*=.029) were significant influencing factors, and the explanatory power was 30% (R^2^=.30, adjusted R^2^=.26, *p*<.001).

## Discussion

This study aims to investigate the sense of calling, organizational commitment, job stress, nursing work environment, and patient safety nursing activities of nurses working in the comprehensive nursing care service units during the COVID-19 pandemic, and to identify factors influencing patient safety nursing activities, to provide basic data for improving patient safety nursing activities of nurses in comprehensive nursing care service units. This paper focuses on discussing the major results of the study. It was found that nurses in comprehensive nursing care service units during the prolonged COVID-19 situation had increased workload and job stress, which was related to the pandemic situation, and the overworked state of the frontline nurses underscores the need for social support systems for them, even if it is necessary for patient safety, as suggested in a previous study. The average score for patient safety nursing activities was 4.77, which supports the results of previous studies, but there have been no studies on the comprehensive nursing care service units using the same measure. The high average score for patient safety nursing activities found in this study and previous studies can be interpreted as indicating that there was greater emphasis on patient safety and that nurses in comprehensive nursing care service units are actively practicing patient safety in the context of the COVID-19 pandemic.

The subcategories of patient safety nursing activities were rated highest in falls prevention (4.77 ± 0.42), followed by infection prevention (4.66 ± 0.46), and the lowest was high-risk medication management (4.01 ± 0.76). This is consistent with previous studies that showed a high level of falls and infection prevention activities among nurses in comprehensive nursing care service units [27]. As the use of comprehensive nursing care service units increases with age, with the elderly over 65 years old and over 70 years old being at high risk for falls, and with orthopedics, neurosurgery, and internal medicine being the main departments in hospitals operating comprehensive nursing care service units, which mainly admit patients with reduced mobility [28], it is important for nurses to actively engage in fall prevention activities in order to prevent falls in hospitalized patients who may not have a primary caregiver. Therefore, it is necessary to provide education on fall prevention knowledge for nurses in comprehensive nursing care service units, develop fall prevention programs that consider ward characteristics, and improve the atmosphere by holding nurses responsible in case of fall accidents.

The next highest subcategory of patient safety nursing activities was infection control, which was rated highly because of the strong transmission of COVID-19 infection, resulting in the strengthening of infection control guidelines in medical institutions and emphasis on infection control by medical staff. Nurses in comprehensive nursing care service units are aware of the importance of infection control for the prevention of COVID-19 infection and perform thorough infection control activities, especially because of the characteristics of the ward that require close contact with hospitalized patients. They also closely monitor any small changes in patients while actively sharing information on infection control guidelines with medical staff, patients, and primary caregivers. Therefore, in the context of COVID-19, an environment for discussing changes in infection control guidelines while performing infection control activities, including hand hygiene, should be created, and collaboration is essential to prevent infection control activities from becoming a source of work-related stress. Patient safety nursing activities are an important indicator in the operation of comprehensive nursing care service units, and insufficient support and personnel shortage, as well as improving the organizational atmosphere related to safety reporting, should be addressed to improve the practical abilities of nurses.

Differences in patient safety nursing activities based on general characteristics showed statistically significant differences in comprehensive nursing care service unit work experience, income, COVID-19 nursing anxiety, and institutional evaluation experience. Regarding comprehensive nursing care service unit work experience, the average score for patient safety nursing activities was higher for those with less than one year of experience and one to two years of experience. This is contrary to a study suggesting that patient safety nursing activities improve with increasing experience due to greater clinical experience and responsibility. However, there is a need for repeated studies as a previous study conducted on nurses in university hospitals and small hospitals showed that those with less than one year of work experience had higher awareness of patient safety culture than those with more experience.

In this study, 36% of nurses working in the comprehensive nursing care service unit had less than three years of experience. This finding reflects the ongoing expansion of the comprehensive nursing service system. It was noted that relatively inexperienced nurses performed patient safety nursing activities while conducting regular patient rounds.

As for income, the results of this study differed from a previous study, which showed that nurses with income levels below 2 million won had higher patient safety nursing activity scores than those with income levels between 2.01 and 2.5 million won. On the other hand, it could also be due to the different characteristics of working hours in the comprehensive nursing care service unit, as there are nurses who work in three shifts, day or night shift in the same unit, which results in different allowances and salaries. Regarding COVID-19 anxiety, a group of nurses who did not experience anxiety symptoms when thinking about COVID-19 patients performed better patient safety nursing activities, indicating that job insecurity has a negative impact on safe behavior. In addition, a study conducted during the COVID-19 pandemic showed that nurses' anxiety about COVID-19 had negative effects on physical activity.

As for institutional evaluation experience, since the certification evaluation system for medical institutions emphasizes the importance of patient safety, nurses with institutional evaluation experience have increased knowledge and attitudes about patient safety and are making efforts to comply with institutional patient safety regulations to prepare for evaluations. Therefore, in order to enhance patient safety management activities of nurses in small and medium-sized hospitals' comprehensive nursing care service units, it is necessary to establish patient safety departments and standardize guidelines and expand the scope of certification evaluations within small and medium-sized hospitals, as the number of safety management departments in these hospitals is relatively low compared to larger medical institutions.

There was a positive correlation between patient safety nursing activities, sense of calling, organizational commitment, and nursing work environment among the study participants, while job stress showed a negative correlation. Emotional organizational commitment influences safe behavior and is supported by research that shows a correlation between emotional r [29, 30]. To secure nursing personnel for patient safety, it is necessary to establish a fair compensation system for existing nurses and prevent the loss of personnel, creating a cooperative atmosphere among hospital members.

Factors influencing patient safety among nurses in comprehensive nursing care service units were analyzed using hierarchical multiple regression analysis. According to Model 3, the significant influencing factors were the length of experience in comprehensive nursing care service, income, anxiety on COVID-19, institutional evaluation experience, sense of calling and job stress. However, considering the high turnover rate among nurses with 3 to 5 years of experience in comprehensive nursing care service units [31], it is necessary to establish an appropriate compensation and welfare system for experienced nurses to enhance their patient safety performance. To reduce COVID-19 anxiety among nurses, hospital-level programs to develop and implement anxiety reduction strategies, such as psychological therapy, should be considered [32].

While sense of calling has been identified as the most significant variable in patient safety in the nursing field, there are limitations to discussing the influence of sense of calling and patient safety nursing activities due to the scarcity of previous studies that have applied research from other fields [33]. However, examining similar studies shows that nurses perform their duties to the best of their ability for the sake of patients during disasters, and in the case of the COVID-19, it is inferred that they exhibit a higher sense of professional responsibility for patient safety. Sense of calling is an important factor in nursing workforce management, as it enhances the organizational competitiveness of nurses and serves as a catalyst for achieving organizational performance [34, 35]. Nursing is a profession where a sense of calling is more important than any other profession, and because of the high employment rate for nurses, practical motivations outweigh sociocultural aspects such as volunteering and sacrifice. Therefore, it is necessary to reflect educational curricula on sense of calling, which involves doing socially meaningful work from undergraduate education, and mentoring programsfor nurses [36, 37].

The job stress investigated in this study refers to effort-reward imbalance (ERI), which is characterized by an imbalance between the effort invested and the rewards received within an organization. This situation creates an atmosphere where members are not fully appreciated, promotion prospects are poor, and job positions and income levels are inadequate for their education and career levels. In small- and medium-sized hospitals, opportunities to enhance expertise are limited, and there are insufficient opportunities for capacity development [38]. Inappropriate treatment and compensation can lead to job stress among nurses; therefore, career management systems for nurses in small- and medium-sized hospitals should be implemented, and opportunities for skills development should be provided. There is a correlation between job stress and increased workload before and after the COVID-19 outbreak. Due to the recent spread of COVID-19 infection, nurses have been required to make additional efforts in tasks such as working at screening clinics, managing treatment centers, and monitoring home care patients, in addition to their regular duties [39]. Therefore, it is necessary to examine whether there are discrepancies in salary due to COVID-19-related work between medical institutions and whether nurses are under excessive pressure from social pressure.

Additionally, a study [40] targeting nurses in the comprehensive nursing care service units reported that some nurses tried to hide infection exposure due to reprimands. This indicates a lack of protective measures for nurses in handling exposure to infections that may occur while caring for COVID-19 patients. Inappropriate treatment is a factor that hinders infection prevention nursing intervention related to patient safety; therefore, it is necessary to improve the organizational culture to prevent individual mistakes when reporting safety concerns.

In contrast, organizational commitment, and nursing work environment, which were found to have significant correlations in previous studies [41] as factors influencing patient safety nursing activities, did not emerge as significant factors in this study. Regarding organizational commitment, a regression analysis was conducted again with the inclusion of the highly correlated emotional organizational commitment sub-factor, but the results remained non-significant. In previous studies, the proportion of organizational commitment among those over 30 years old was 39% and 34.2%, respectively, but in this study, those in their 20s accounted for the highest percentage at 44.2%, indicating different results. Therefore, further research is needed to investigate the factors influencing organizational commitment and patient safety management behavior. As for the nursing work environment, the lack of patient safety manuals and the subjectivity of the tool measurement concept suggest the need for repeated studies using direct variables.

### Nursing implications

This study aimed to measure the sense of calling and identify factors affecting patient safety activities among comprehensive nursing care service unit nurses during the COVID-19 pandemic and contribute to developing basic data that can be used in developing safety nursing activity indicators. Additionally, as a strategy to enhance patient safety nursing activities among comprehensive nursing care service unit nurses, it is suggested that a mentoring relationship should be developed and applied to increase their sense of calling and to reduce job stress.

Moreover, an intervention study should be conducted to examine whether the imbalance between the efforts and rewards of comprehensive nursing care service unit nurses affects patient safety nursing activities. To improve patient safety nursing activities among comprehensive nursing care service unit nurses, it is recommended to establish a reward system for nurses, measure excessive effort related to intertwined nursing duties, and verify the effects of reducing job stress through medical institution support systems.

### Limitations

First, the study was limited in scope to nurses working in integrated nursing and care service wards in small and medium-sized hospitals in Seoul and some areas of the Gyeongin region with at least one year of clinical experience, and the survey was conducted online during a period when the number of COVID-19 confirmed cases was increasing. Therefore, caution is needed in generalizing the results even within the selected sample of nurses who responded faithfully to the online survey. Secondly, the instruments used in this study were created before the COVID-19 situation, so there may be differences in content from the sense of mission, organizational commitment, job stress, and nursing work environment of nurses in integrated nursing and care service wards during the current situation. Finally, since the infection control measures for COVID-19 vary by institution among nurses working in integrated nursing and care service, there may be differences in the COVID-19 nursing experiences of the subjects.

## Conclusion

This descriptive survey aimed to gather fundamental information about the factors that affect patient safety nursing activities among nurses in comprehensive nursing care service units, including sense of calling, organizational commitment, job stress, and nursing work environment. The study focused on identifying the factors that contribute to patient safety nursing activities among nurses in comprehensive nursing care service units during the COVID-19 pandemic. The study employed the job demands-resources model as a conceptual framework. Among the general characteristics, factors such as work experience in comprehensive nursing care service unit, income, anxiety levels in caring for COVID-19 patients, sense of calling, and job stress were identified as influencing patient safety management activities. Higher levels of education, income, and sense of calling were associated with increased engagement in patient safety management activities. Conversely, lower experience in comprehensive nursing care service unit, lower anxiety levels in caring for COVID-19 patients, and reduced job stress were also linked to higher levels of patient safety nursing activities. To enhance patient safety management activities, it is necessary to develop a sense of calling education program, improve nursing work systems tailored to the demands of the COVID-19 situation, and establish a fair compensation system.

## Implications for future research and practice

First, future research could explore innovative approaches that can strengthen nurses' sense of calling, through mentorship and institutional recognition. Second, a mediation study should be conducted to determine if there is an imbalance between the efforts and rewards of nurses in comprehensive nursing care service units that affects patient safety nursing services. Additionally, by quantifying the extent of excessive effort and its implications for patient safety, measuring excessive effort in interconnected nursing tasks requires the development of reliable metrics and tools that accurately capture the workload and stressors experienced by nurses in comprehensive care settings. Third, further research should be conducted with a broader range of participants from various medical institutions as the study only included nurses working in small and medium-sized hospitals in some regions. Finally, subsequent research should be conducted to identify new variables related to patient safety management activities that may have an impact beyond the sense of calling and job stress among nurses in comprehensive nursing care service units.

## Data Availability

The data that support the findings of this study are available from the authors Korea, upon reasonable request.
